# JNK inhibitor IX restrains pancreatic cancer through p53 and p21

**DOI:** 10.3389/fonc.2022.1006131

**Published:** 2022-12-07

**Authors:** Jingwei Shi, Xing Yang, Qi Kang, Jian Lu, Maximilian Denzinger, Marko Kornmann, Benno Traub

**Affiliations:** ^1^ Department of Cardiothoracic Surgery, Nanjing Drum Tower Hospital, The Affiliated Hospital of Nanjing University Medical School, Nanjing, China; ^2^ Department of General and Visceral Surgery, Ulm University Hospital, Ulm, Germany

**Keywords:** pancreatic cancer, c-Jun N-terminal kinase, JNK inhibitor IX, cell cycle arrest, G2 arrest

## Abstract

Novel treatment options for pancreatic cancer are desperately needed. De-regulated kinases can be regularly detected in pancreatic cancer. Multiple pathway inhibitors were developed to exploit these features, among them selective inhibitors of the c-Jun N-terminal kinase isoforms 1 and 2 (JNK1 and 2). We evaluated the effectiveness of four different JNK inhibitors on pancreatic cancer cell lines. Cell mobility and migration were evaluated in scratch assay and Boyden chamber assay. Mechanism of cell death was analyzed *via* apoptosis assays in FACS and immunoblotting as well as cell cycle analysis *via* FACS, and qPCR. JNK2 knockout cells were generated using siRNA transfection. Among the inhibitors, JNK inhibitor IX (JNK-in-IX), designed as specific inhibitor against JNK2 was proven highly effective in inhibiting cell growth, mobility and migration. We were able to show that JNK-in-IX caused DNA damage resulting in G2 arrest mediated through p53 and p21. Interestingly, JNK-in-IX acted independently of its primary target JNK2. In summary, JNK-in-IX was shown highly effective in pancreatic cancer. This study underlines the need for modeling systems in testing therapeutic options as JNK2 was previously not indicated as a potential target.

## Introduction

The devastating numbers of pancreatic cancer (PC) are well known around researchers and clinicians: fourth leading cause of cancer-related mortality, estimated to rise to second position within next decade (1, 2); no improvement in mortality rates over the last decade, unlike other malignancies (1); failure of early detection and severe side-effects in treatment (3).

In search for alternative treatment options, researchers have focused on signaling pathways that were found altered in PC. While mutant KRAS is the key driver of PC, it has yet been mostly undruggable (4). Addressing signaling pathways downstream of KRAS has thus become of interest, but the combined inhibition of major downstream pathways, the PI-3-/Akt-kinase and the MAPK pathways has failed in patients (5). Thus, a more thorough understanding of these pathways is needed.

The c-Jun N-terminal kinases 1, 2, and 3 (JNK1/2/3) form a major subgroup among MAP kinases, and, together with the p38 kinase are grouped as stress activated kinases (6). This is due to their physiologic role in the cellular response towards stressors like proliferation and metabolic stress as well as the response towards exogenic triggers like chemotherapeutic drugs and irradiation (7). These variable stimuli are transduced into a cellular response carried out by transcription factors, mainly c-Jun (8). Thereby, the central role of JNKs becomes especially evident in PC, as RAS-induced oncogenesis is dependent on Jun phosphorylation (9, 10).

Still, it is yet to determine if the JNK isoforms act as tumor promotors or suppressors. Multiple well-designed studies evaluated the role of JNK1 and 2 in cellular transformation and have offered evidence for both malignant transformation (11–15) and tumor suppression (15–17). In PC we recently showed a tumor promoting phenotype after JNK2 knockdown, while JNK1 knockdown seemed to reduce the cellular malignant potential (18).

Studies in pharmacological targeting of JNK can be biased by inhibiting isoforms nonspecifically as well as off-target effects and compensatory mechanisms, e.g in the p38 pathway (6). However, isoform specific JNK inhibitors were developed recently and the inactivation of JNK1 sensitized PC cells towards FOLFOX treatment (19).

This study aimed to determine value and mechanism of JNK inhibition in PC. We used novel isoform specific inhibitors of JNK to test their efficacy in PC cell lines. Contrary to previous findings we showed the highest efficacy for JNK inhibitor IX (JNK-in-IX), designed as specific inhibitor of JNK2. JNK-in-IX caused a G2 arrest through upregulation of p21 and p53 phosphorylation and markedly reduced cell migration, but surprisingly seemed to act independently of JNK2.

## Materials and methods

### Cell lines and cell culture

Human pancreatic cancer cell lines AsPC-1 (RRID : CVCL_0152), BxPC-3 (RRID : CVCL_0186), MIA PaCa-2 (RRID : CVCL_0428), and PANC-1 (RRID : CVCL_0480) were purchased from the American Type Culture Collection (ATCC, Manassas, USA). AsPC-1 was cultured in RPMI (Roswell Park Memorial Institute medium). BxPC-3 was cultured in 50% RPMI and 50% DMEM (Dulbecco’s Modified Eagle’s Medium). MIA PaCa-2 and PANC-1 were cultured in DMEM. All mediums were supplemented with 10% fetal calf serum (FCS), 1% Penicillin (10,000 U/ml)/Streptomycin (10,000 μg/ml). Cells were maintained in 100 mm cell culture dishes and in a monolayer culture at 37°C in humidified air with 5% CO2. Regular mycoplasma testing was carried out.

### Human pancreatic cancer organoids

Human pancreatic cancer organoids were generated and maintained as described before (20). Organoid work was performed in cooperation with the Core Facility Organoids of the University Hospital of Ulm. Organoid generation and analysis were approved by the independent ethics committee of the University of Ulm (approval number 72/19) and written informed consent was obtained from patients before collecting the samples.

### Dose response of pancreatic cancer cell lines to JNK inhibitors and chemotherapeutic drugs

JNK inhibitors SP600125, AS602801, JNK-in-IX and Licochalcone, supplied by Selleck Chemicals GmbH (Planegg, Germany), were dissolved and aliquoted according to manufacturer’s instructions. The combination regimen of FOLFIRINOX is composed of 5-FU (Sigma Aldrich, Taufkirchen, Germany), Oxaliplatin (Sigma Aldrich), and SN-38 (active compound of Iriontecan, Sigma Aldrich) in ratios of 5-FU: Oxaliplatin: SN-38 = 80.95: 0.80: 1 and Gemcitabine (Sigma Aldrich) – Paclitaxel (Sigma Aldrich) (Gem-Pac) in ratios of Gemcitabine: Paclitaxel = 1: 0.04. PC cells were seeded at a density of 500 cells/well in 384-well plates. Cells were allowed to adhere for 24 h. Then, drugs were dispensed using the Tecan D300e (Tecan Deutschland GmbH, Crailsheim, Germany) with titration concentrations ranging from 0.0001 µM to 50µM for chemotherapeutic drugs and from 0.01 µM to 50 µM for inhibitors, while DMSO was normalized to the highest concentration (0.5%, v/v). Synergy mode supplied by the D300e was used to dispense the combination of chemotherapies and JNK inhibitors. 5 days after treatment, cell viability was assessed by CellTiter-Glo^®^ Luminescent Cell Viability Assay (Promega GmbH, Walldorf, Germany) following the manufacturer’s instructions.

Human pancreatic cancer organoids were dissociated into single cells and 500 cells per well were seeded in triplicate in 1µl Matrigel domes in 384-well plates and 25µl of organoid growth medium was added. After 24 h, 25µl of organoid growth medium containing the drugs was added. 10 concentrations per drug were used. Cell viability was assessed using CellTiter-Glo^®^ 3D Cell Viability Assay (Promega GmbH) after 5 days of treatment.

Data were analyzed using GraphPad Prism 8.0.1 (GraphPad Software, San Diego, USA). Synergetic effects of chemotherapies and JNK inhibitors was evaluated by the SynergyFinder 2.0 platform (21).

Effect of V-ZAD-FMK (50 µM, Selleck Chemicals) on cell growth with or without JNK-in-IX treatment was also determined following the above procedures.

### Cell migration assay

Wound healing assay was performed to examine cell movement and Boyden chamber assay was used to determine cell migration as described before (22). In brief, the gaps of wounds scratched by sterile 200 µl tips on confluent cells in a monolayer were measured. Gap distances were quantitatively evaluated by ImageJ 1.8.0 (National Institutes of Health, Bethesda, USA). In the Boyden chamber assay, migratory cells were stained with DAPI (Sigma Aldrich) and counted by ImageJ 1.8.0. In both assays, the intervention group was treated with JNK-in-IX for 48 h using the previously determined cell line specific concentrations matching the IC50: 0.409 µM for AsPC-1, 0.220 µM for BxPC-3, 0.071 µM for MIA PaCa-2 and 0.066 µM for PANC-1. Wound healing rate and number of migratory cells were analyzed and compared between the treated and untreated groups using GraphPad Prism 8.0.1.

### Giemsa stain assay

Giemsa staining was performed as described before (22). PC cells stained by Giemsa stain (Sigma Aldrich) were photographed under a light microscope at 20x and 40x magnification. Afterwards, cell morphology was observed.

### Western blot analysis

Western blot was performed as described before (22). Primary antibodies were used as follows: Bcl2 (1:200, Cell Signalling Technology (Frankfurt am Main, Germany)), BAX(1:1000, Cell Signalling Technology), c-Jun(1:100, Santa Cruz Biotechnology (Dallas, USA)), p-c-Jun(1:100, Santa Cruz Biotechnology), Lamin B1 (0.1 µg/ml, Abcam). β-actin(1:5000, Sigma Aldrich) and GAPDH (1:5000, Sigma Aldrich) act as the internal control. Images were acquired by FUSION FX (Vilber Lourmat Deutschland GmbH, Weinheim, Germany).

### Flow cytometry

Apoptosis was analyzed in the Annexin V assay using the Annexin V-FITC Kit (Miltenyi Biotec, Bergisch Gladbach, Germany) following the manufacturer’s instructions. 10^6^ of PC cells with or without JNK-in-IX incubation for 48 h were harvested, mixed with 10 μl of Annexin V-FITC and incubated for 15 min at room temperature. After washing, the cell pellet was resuspended in 500 μl of binding buffer. 5 μl of PI (Propidium Iodide) solution was added immediately prior to flow cytometry using MACSQuant^®^ X Flow Cytometer (Miltenyi Biotec).

Cell cycle was analyzed by performing PI staining (Sigma Aldrich). 10^6^ PC cells were harvested and fixed in cold 70% ethanol. After fixation for 2 h, the cell pellet was rinsed and resuspended in staining solution containing 50 μg/ml of PI. Data were acquired by flow cytometry.

For flow cytometric expression of JNK1 and JNK2 protein levels, 10^6^ PC cells were harvested and blocked in FC-block solution (Miltenyi Biotec, Bergisch Gladbach, Germany) for 20 min. 100 ul of single cell suspension containing 10^6^ cells of interest were aliquoted and incubated in 300 µl of 2.7% paraformaldehyde for 30 min. After being rinsed, cells were resuspended in 500 of µl ice-cold permeabilizing solution and centrifuged at 350 x g at 4°C for 8 min and the supernatant was discarded. Then, cells were stained by AF647-conjugated antibodies (anti-JNK1 antibody and anti-JNK2 Antibody, 1:50, Santa Cruz Biotechnology (Dallas, USA)). Cells resuspended in 300 µl of FACS buffer were subjected to flow cytometry.

Data were analyzed by FlowJo_v10.6.1 (FlowJo LLC, Ashland, USA).

### Immunocytochemistry/Immunofluorescence (ICC/IF)

Cells were cultured on glass coverslips until 50-80% confluence. A control and drug-treated group were set up, and cells were treated with or without JNK-in-IX for 48 h at beforementioned concentrations. After rinsing with PBS, cells were fixed with 4% paraformaldehyde for 20 min and permeabilized by incubation with 0.2% Triton for 10 min at room temperature. Cells were incubated with 5% BSA to block nonspecific binding and then incubated with phospho-histone H2A.X (1:400, Cell Signaling Technology) overnight at 4°C. After rinsing, cells were incubated with Alexa Fluor Plus 594-labeled anti-rabbit secondary antibody (10 µg/ml, Invitrogen) for 1 h at room temperature. Nuclear staining was performed with DAPI (2µg/ml, Sigma-Aldrich). Representative fluorescence photographs were taken using Axio Observer 7 (Zeiss) at 40x magnification.

### Quantitative real-time PCR

Quantitative real-time PCR (qPCR) was used to determine the target gene expression on mRNA level. Total RNA in cell lysates was extracted using Monarch^®^ Total RNA Miniprep Kit (New England BioLabs, Ipswich, USA). The concentrations of purified total RNA were detected using the QIAxpert (QIAGEN, Hilden, Germany). cDNA was synthesized using the AffinityScript Multiple Temperature cDNA Synthesis Kit (Agilent Technologies, Santa Clara, USA). The expression of target genes (JNK1, JNK2, CDK1, CCNB1, CDC25C1, PLK1, CHEK1, CDKN1A, p53, and Wee1) was verified by qPCR using SYBR-Green Master Mix (New England BioLabs) with LightCyter^®^480 II (Roche Life Science, Mannheim, Germany). Experiments were performed following the manufacturers’ protocols. Primers were supplied by QIAGEN and are listed in the **Supplementary Table S1**.

### siRNA transfection

Each cell line was seeded into 2 ml of complete medium in triplicate in a 6-well plate and cultured until cells were at 50-70% confluence at the time of transfection. For transfected cells, 25 pmol of MAPK-9 RNAi (Silencer Select siRNA, ID: S11159, Catalog 4390824 (Thermo Fisher Scientific, Waltham, MA, USA)) or 25 pmol of Silencer Select Negative Control No.1 siRNA (Catalog 4390843, Thermo Fisher Scientific) was dissolved in 150 µl of Opti-MEM^®^ Medium (Catalog 31985070, Thermo Fisher Scientific). Diluted Lipofec-tamine™ RNAiMAX (Catalog 13778100, Thermo Fisher Scientific) as transfection reagents were separately added. Lipofectamine and siRNA solutions were mixed gently and incubated for 5 min at room temperature prior to adding into each well. Cells were passaged 24 h after transfection, and transfection efficiency was verified by qPCR.

In order to determine cell growth, 1000 of wildtype, JNK-2 knockdown and control transfected pancreatic cancer cells were seeded into each well of a 384-well plate containing 50 µl of medium. JNK-in-IX was added after cell adherence with the concentrations previously mentioned. 48 h and 96 h after JNK-in-IX treatment, viability of wildtype, JNK-2 knockdown or control transfected cells in four cell lines was detected as described above.

### Human Phospho-Kinase array (proteome profiler)

To analyze the involved pathways after JNK-in-IX treatment, phosphorylation of relevant kinases and transcription factors were detected by using the Human Phospho-Kinase Array Kit (ARY003C, R&D Systems, Inc, Minneapolis, USA) following manufacturer’s instructions. Briefly, PC cells with or without JNK-in-IX incubation for 48 h were lysed and subjected to the array membranes. Images were acquired by FUSION FX. Mean pixel density was analyzed by ImageJ 1.8.0 and GraphPad Prism 8.0.1.

### Statistics

GraphPad Prism 8.0.1 was used for statistical analysis. To evaluate the significance of differences among groups, statistical methods including t test, paired t test, Tukey’s multiple comparisons test and uncorrected Fisher’s LSD were used. P values less than 0.05 are taken as significant and are shown as follows: ns p > 0.05, * p < 0.05, ** p < 0.01, *** p < 0.001 and **** p < 0.0001.

## Results

We have previously demonstrated the role of the JNK isoforms (JNK1 and JNK2) in PC. A potential therapeutic role has been outlined by others, even more since the development of isoform-specific inhibitors. These results have prompted us to investigate the potential use of pharmacological targeting of JNK in PC.

### Pancreatic cancer cell survival is reduced by JNK inhibitors, most significantly by the JNK2-specific inhibitor JNK inhibitor IX

**Figure 1:**
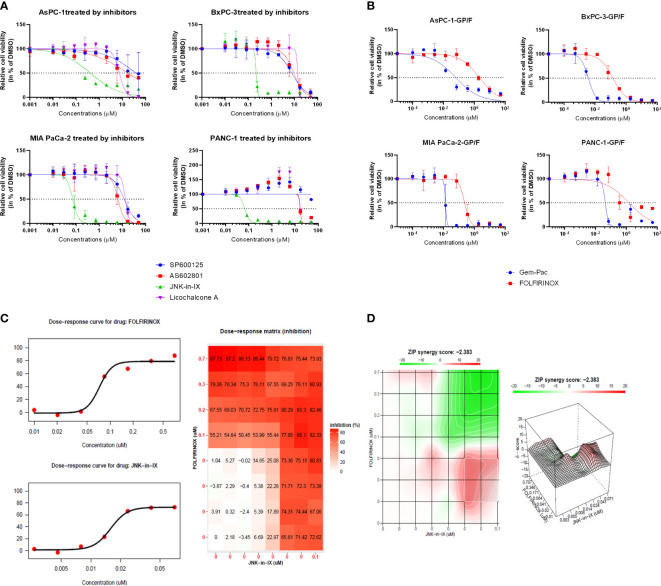
Targeting pancreatic cancer cells. **(A, B)** 500 cells per well were seeded in 384-well plates containing 50 μl of complete medium. 24 h after seeding, increasing concentrations of JNK inhibitors **(A)** and chemotherapeutics **(B)** dissolved in DMSO were added in triplicate using the Tecan D300e Dispenser. DMSO was normalized to the highest concentration (0.5%, v/v). Cell viability was evaluated 5 days after treatment using CellTiter-Glo. Results shown are means of 3 independent experiments. **(C, D)** Combination treatment with JNK-in-IX and FOLFIRINOX in MIA PaCa-2. Data were calculated and visualized using SynergyFinder 2.0. Results are means of 3 independent experiments. With a ZIP score of less than -10, the interaction between two drugs is likely to be antagonistic; from -10 to 10: the interaction is likely to be additive, and for scores larger than 10, the interaction is likely to be synergistic. GP: Gemcitabine-Paclitaxel (Gem-Pac): the combination of Gemcitabine and Paclitaxel with the ratio of 1:0.04 (c/c); F FOLFIRINOX: the combination of SN-38, Oxaliplatin and 5-FU with the ratio of 1:0.8:80.95 (c/c/c).

In order to test the response of human PC cell lines to JNK inhibition, the efficacy of two pan-JNK inhibitors (SP600125 and AS602801) as well as the JNK1-specific inhibitor Licochalcone A and the JNK2-specific inhibitor JNK inhibitor IX (JNK-in-IX) were tested. The dose-response curves demonstrated in **Figure 1A** clearly show that no difference between JNK1 specific inhibition and pan-JNK inhibition can be observed. However, JNK-in-IX was proven highly efficient with IC50 values of about 0.1 µM across all cell lines (**Figure 1A**, **Supplementary Table S2**).

We next examined the combination therapy of JNK-in-IX and the standard chemotherapeutic regimen for human pancreatic cancer cell lines, FOLFIRINOX and Gemcitabine-Paclitaxel (Gem-Pac). The dosages used for combination treatment matched the *in vivo* situation with concentrations of drugs at the ratios of Gemcitabine: Paclitaxel = 1: 0.04 and 5-FU: Oxaliplatin: SN-38 = 80.95: 0.80: 1 (23). Both Gem-Pac and FOLFIRINOX were effective in all tested cell lines (**Figure 1B**). We evaluated the combination treatment by using SynergyFinder 2.0 in order to discriminate between additive, synergistic or antagonistic effects (**Figures 1C, D**). Data was evaluated using the ZIP (Zero Interaction Potency) model (**Supplementary Table S3**) where drugs are assumed to be non-interacting and differences in the dose-response curve can be evaluated (21). Thereby, we were able to demonstrate that no synergistic effect can be observed in the combination treatment of Gem-Pac or FOLFIRINOX with JNK-in-IX. For AsPC-1, the combination of FOLFIRINOX and JNK-in-IX even seemed antagonistic (**Supplementary Table 2**).

**Figure 2:**
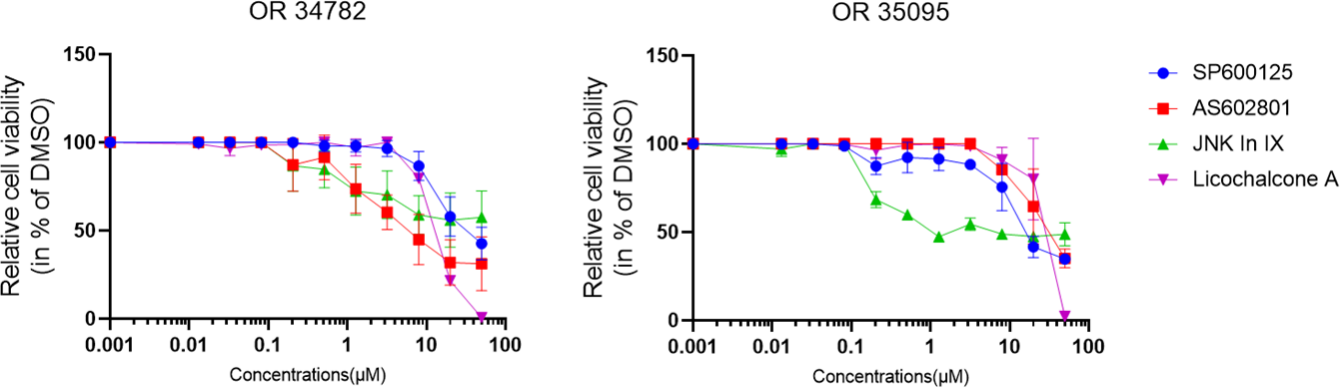
Human PC organoid treatment. Human organoids OR34782 and OR35095 were generated from PC specimen. 500 cells per well were plated in 1µl of Matrigel and cultured in 25µl Organoid growth medium. After 24 h, another 25 µl of Organoid medium was added including the indicated inhibitors. Results are shown as means of 3 (OR34782) and 2 independent replicates (OR35095).

We also tested our findings in 2 organoid lines derived from PC specimen. The effects were less pronounced, but JNK-in-IX was still effective in both organoid lines (**Figure 2**).

### JNK-in-IX reduces pancreatic cancer cell migration

After JNK-in-IX was shown to be highly effective in suppressing cell survival of PC cells, its effect on cell behavior was examined next. First, it became obvious, that JNK-in-IX treatment leads to morphological changes with enlarged cells including larger nuclei as demonstrated *via* Giemsa staining (**Supplementary Figure S1**). Therefore, we evaluated cell mobility and migration next: AsPC-1, BxPC-3, MIA PaCa-2 and PANC-1 were treated with 0.409 µM, 0.220 µM, 0.071 µM and 0.066 µM of JNK-in-IX, corresponding to the previously determined IC50 for each cell line. In previous findings from our group, the reduced expression of JNK2 led to enhanced cell migration (18).

**Figure 3:**
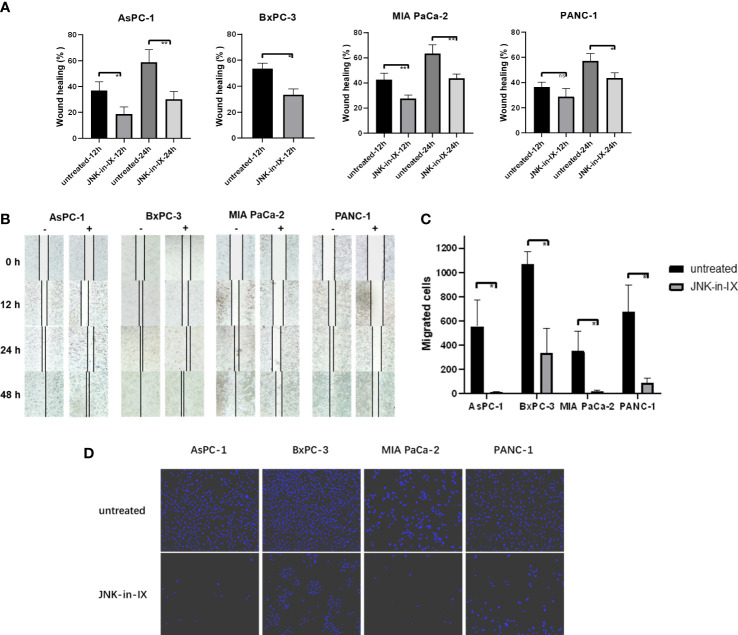
Effect of JNK-in-IX on pancreatic cancer cell mobility and migration. **(A)** Cell movement in the wound healing assay. Results are shown as wound healing distance (in % of 0 h) after 12 h and 24 h and are means of 5 separate experiments. The wound healing rate of BxPC-3 at 24 h is not shown as the wound margins were highly irregular. **(B)** Representative areas of wound gaps at 0 h, 12 h, 24 h and 48 h at 2.5× magnification. **(C, D)** Modified Boyden chamber assay. Results are shown as number of the migrated cells within 24 h and are means of 5 separate experiments **(C)** with representative areas of migrated cells at 10× magnification **(D)**. ns p>0.05, * p< 0.05, ** p<0.01.

In contrast to these findings, by treatment with JNK-in-IX, pancreatic cancer cell migration was profoundly reduced. The scratch assay showed prolonged wound closure rates of about 20% across all cell lines (**Figures 3A, B**). Even more strikingly, the cellular migration ability was nearly completely abolished in the modified Boyden chamber assay (**Figures 3C, D**). These findings were replicable in all 4 biological replicates.

### JNK-in-IX induced cell death

These results indicate that JNK-in-IX can strongly reduce oncogenic hallmarks of PC cells and we next sought to examine the mechanism of action.

**Figure 4:**
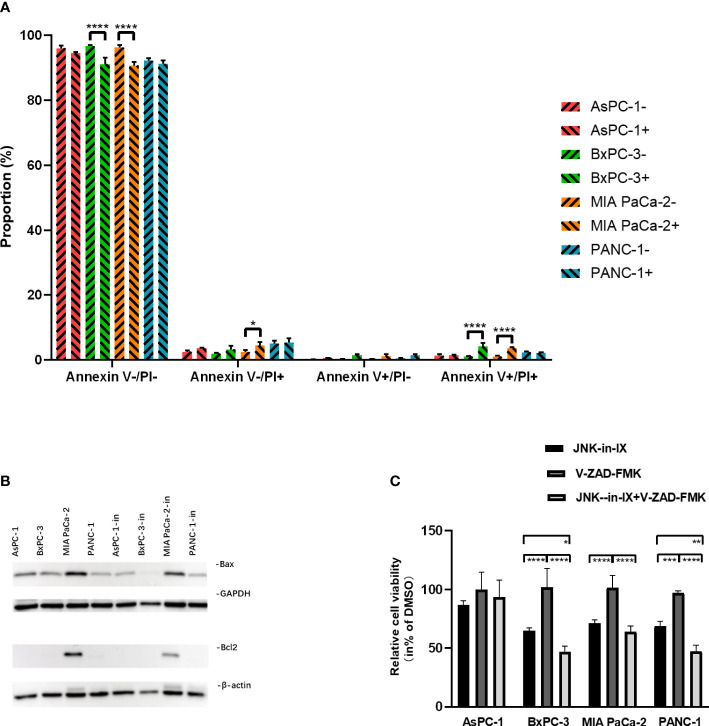
Effect of JNK-in-IX on apoptosis. **(A)** After incubation with JNK-in-IX (AsPC-1: 0.409 µM, BxPC-3: 0.220 µM, MIA PaCa-2: 0.071 µM, and PANC-1: 0.066 µM) for 48 h, 10^6^ cells were harvested and stained by Annexin V and PI. Data were analyzed by flow cytometry. Viable cells are stained Annexin V- / PI-, apoptotic cells are stained Annexin V+ / PI-, and dead cells are stained Annexin V+ / PI+. Only a slight increase in apoptotic cells was observed. Results are shown of 3 independent experiments. **(B)** Western blot analysis of Bax and Bcl2 after JNK-in-IX treatment. GAPDH and β-actin were used as internal control. **(C)** Pancreatic cancer cell lines treated by JNK-in-IX and V-ZAD-FMK. Percentage of viable cells compared to untreated control are shown. Data are expressed as mean ± SEM. of at least four independent experiments. *p < 0.05, **p < 0.01, ***p <; 0.001, ****p < 0.0001.

PC cells were treated with JNK-in-IX using the same concentrations as above (corresponding to IC50) for 48 h. We first evaluated apoptosis-related cell death. By using Annexin V/Propidium Iodide (PI)-staining, we were able to demonstrate, that JNK-in-IX only slightly increased the proportion of apoptotic Annexin V+/PI- cells. Proportions of dead cells (Annexin V+/PI-) increased significantly in BxPC-3 and MIA PaCa-2. However as detached dead cells are mostly lost during cell harvest, these results need to be interpreted cautiously (**Figure 4A**).

After JNK-in-IX treatment, expression of pro-apoptotic Bax was decreased in all cell lines. The anti-apoptotic Bcl-2 was only found highly expressed in MIA PaCa-2 and was also decreased after JNK-in-IX treatment (**Figure 4B**). These results indicate, that JNK-in-IX only slightly increases apoptosis in PC cells and the observed effect of reduced cell survival appears to be mostly apoptosis independent. We were able to verify these results by using the pan-caspase inhibitor Z-VAD FMK which can prevent apoptosis-related cell death. Corresponding to previous results, caspase-inhibition through Z-VAD FMK was not able to reverse JNK-in-IX induced cell death (**Figure 4C**).

**Figure 5:**
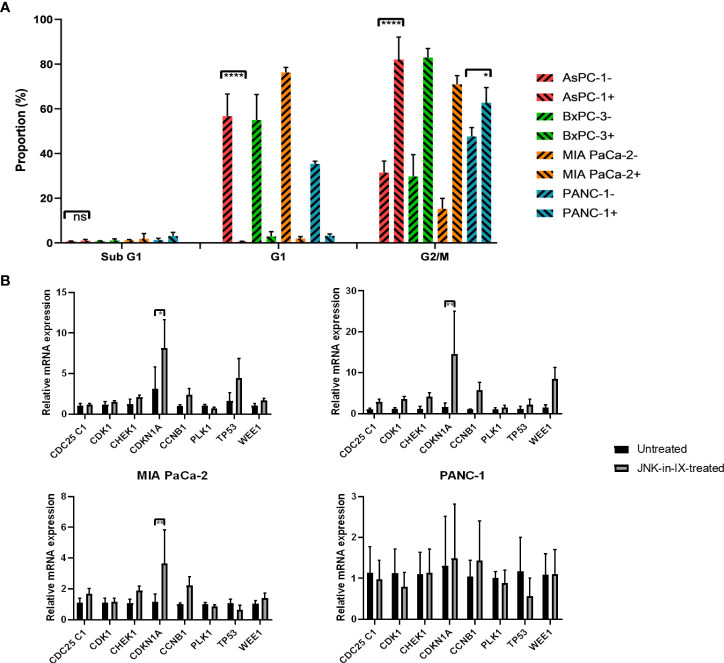
**(A)** Cell cycle analysis. After treatment with JNK-in-IX in the previously determined concentrations for 48 h, 10^6^ PC cells were harvested, fixed, and stained with PI staining buffer. Afterwards, data were acquired using a flow cytometer. Cell cycles were normalized to the untreated control of which proportions are presented. All cell lines show a significant decrease in the G1 phase and a corresponding increase of cells in G2/M phase (p < 0.0001 for all cells except PANC-1, p < 0.05 for PANC-1). **(B)** mRNA expression of cell cycle regulators. Cyclin Dependent Kinase 1 (CDK1), Cyclin B1 (CCNB1), CDC25C1, Polo-like Kinase 1 (PLK1), Checkpoint Kinase 1 (CHEK1), p21 (CDKN1A), p53, and Wee1 expression in pancreatic cancer cells treated by JNK-in-IX was tested by qPCR. Data are shown as fold changes with respect to the untreated group (2-ΔΔCt, mean ± SEM, n = 3). GADPH was used as the internal control. *p < 0.05, **p < 0.01, ****p < 0.0001.

Cell-cycle progression was evaluated next. Again, cells were treated for 48 h with JNK-in-IX in the before mentioned concentrations. There, we were able to detect a strongly increased proportion of cells in the G2 phase, suggesting a G2 arrest through JNK-in-IX (**Figure 5A**).

Expression of Lamin B1, as a nuclear envelope marker was reduced after JNK-in-IX treatment, also indicating that cells fail to successfully undergo mitosis (**Supplemental Figure S2**)

In order to elucidate the mechanism of G2 arrest after JNK-in-IX treatment, we analyzed the expression of key regulators of cell cycle progression. We included pro-mitotic regulators (Cyclin Dependent Kinase 1 (CDK1), Cyclin B1 (CCNB1), CDC25C1, and Polo-like Kinase 1 (PLK1)) as well as restrictors of cell cycle progression (Checkpoint Kinase 1 (CHEK1), p21 (CDKN1A), p53, and Wee1). After treatment with JNK-in-IX, p21 (CDKN1A) was consistently upregulated in all cell lines (**Figure 5B**). Treatment with JNK-in-IX thus leads to a G2 arrest mediated by p21.

### Target effectors of JNK-in-IX

**Figure 6:**
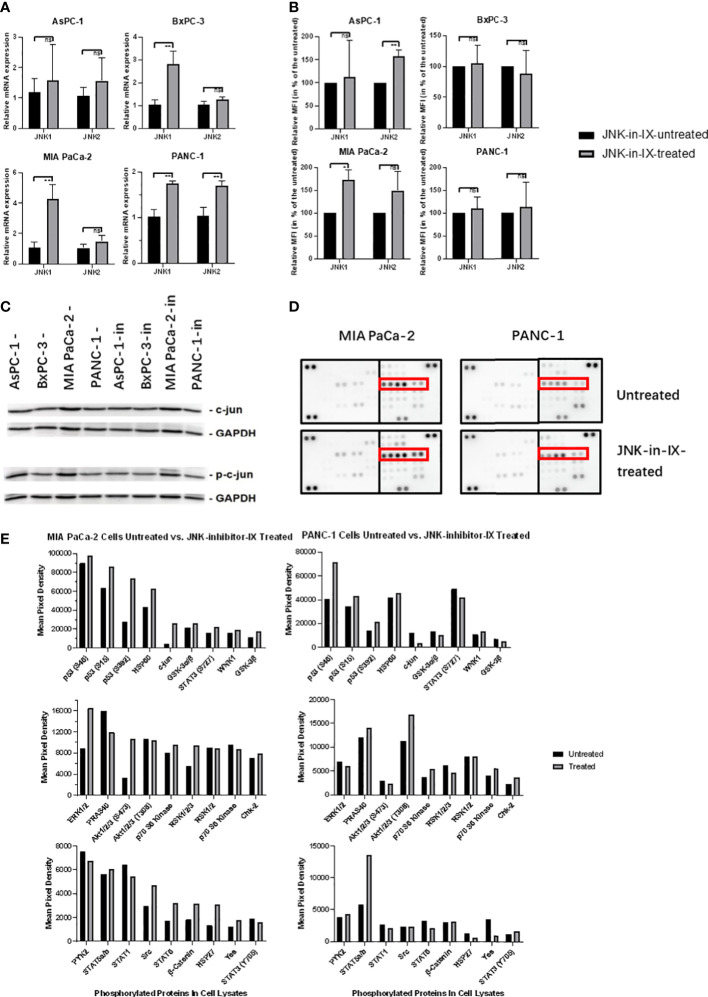
Target effectors of JNK-in-IX. **(A, B)** Relative mRNA expression in qPCR **(A)** and Mean Fluorescence Intensity (MFI) detected by flow cytometry **(B)** of JNK1 and JNK2 with or without JNK-in-IX treatment. Data shown are the means (±SEM) from 3 **(A)** and 4 **(B)** independent experiments. **(C)** Immunoblot analysis of total expression and phosphorylation of c-jun with or without JNK-in-IX treatment. GAPDH acts as internal control. **(D, E)** Human Phospho-Kinase Array. Pictures shown are the blots of respective protein phosphorylation with or without JNK-in-IX treatment, p53 phosphorylation is marked in red **(D)**. **(E)** Quantitative expression of phosphorylated kinases. Data are shown as mean pixel density. ns p>0.05, *p<0.05, **p<0.01.

We next sought to evaluate the effect of JNK-in-IX on the expression of JNK1 and 2. On RNA level, there was a trend towards increased expression of both kinases (**Figure 6A**). We also evaluated protein expression levels using the mean fluorescence intensity in flow cytometry. There, we were only able to observe a slight trend towards increased kinase expression in AsPC-1 and MIA PaCa-2, while expression levels in BxPC-3 and PANC-1 were unaltered (**Figure 6B**). Surprisingly, after evaluation of c-Jun expression and phosphorylation (**Figure 6C**), we did not detect any differences in expression levels of c-Jun and interestingly, also did not detect differences of its phosphorylation.

These results suggested that the effects of JNK-in-IX may be independent of JNK2 activity and c-Jun phosphorylation. Therefore, we used a human phospho-kinase assay to analyze related pathways in MIA PaCa-2 and PANC-1, as those were the most sensitive towards JNK-in-IX (**Figures 6D, E**). Again, we could not detect a consistent difference in c-Jun phosphorylation. However, we could observe a consistent phosphorylation of p53 at serine 15, 46 and 392 in both cell lines.

**Figure 7:**
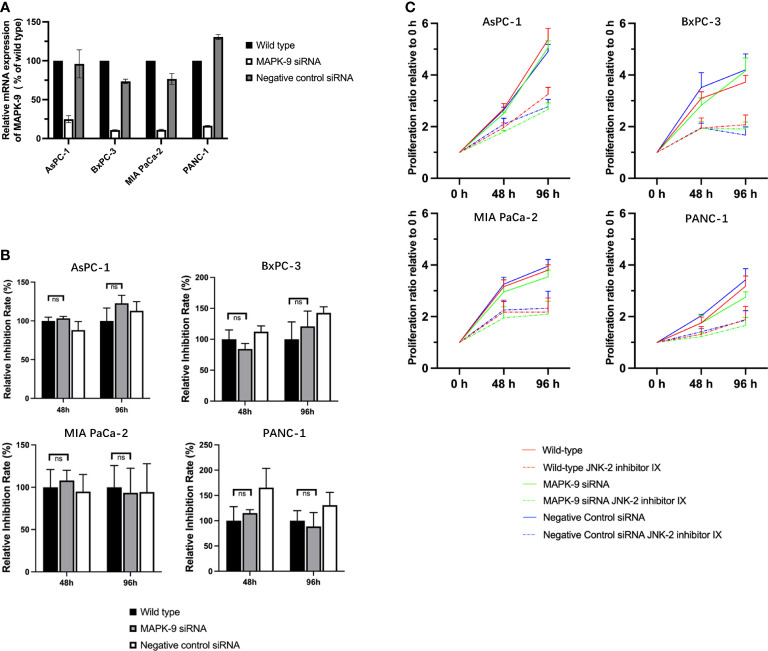
siRNA mediated knockdown of JNK2 does not impact the effect of JNK-in-IX in pancreatic cancer. **(A)** qPCR analysis revealed that siRNA targeting MAPK-9/JNK2 effectively reduces the expression of MAPK-9. Results are shown as means of two replicates in qPCR. **(B)** Quantitative inhibition rate. 2x10^4^/ml of wildtype, control transfected and knockdown pancreatic cancer cells were seeded in quintuplicate into each well of a 384-well plate containing 50 µl of complete medium. JNK-in-IX was added after 24 h with the before mentioned concentrations. Cell viability was measured by CTG after 48 h and 96 h after treatment. The results presented demonstrate the effect of JNK-in-IX on wildtype cells, JNK2 knockdown cells and negative control cells respectively. 100% equals the inhibition rate of wildtype cells at each timepoint. This demonstrates that JNK-in-IX is equally effective irrespective of JNK2 expression. **(C)** Cell growth with or without JNK-in-IX. JNK-in-IX suppressed cell growth after 48 h and 96 h after treatment. Results are shown as proliferation ratio relative to 0 h after JNK-in-IX was added. ns p >0.05.

To verify the hypothesis, that JNK-in-IX acts independently of its designed target JNK2, we used siRNA knockouts of JNK2 in all four cell lines. qPCR confirmed a successful knockdown (**Figure 7A**). We then evaluated cell growth of wildtype cells, control transfected cells and knockdown cells with or without JNK-in-IX treatment using the concentrations previously determined. Again, JNK-in-IX successfully suppressed cell growth in wildtype and control-transfected cells. However, it was equally effective in cells with reduced expression of JNK2 (**Figures 7B, C**). This further strengthened our findings that JNK-in-IX acts independently of its primary target JNK2 in PC.

**Figure 8:**
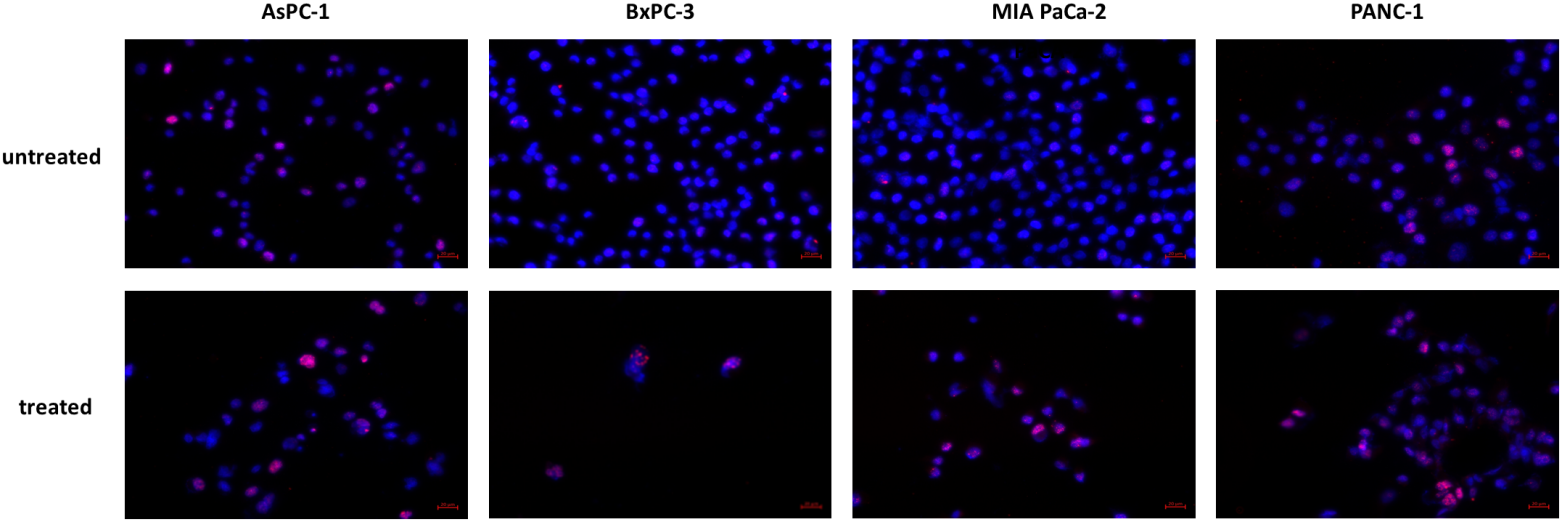
Histone H2A.X phosphorylation. Increased phosphorylation of Histone H2A.X was detected after JNK-in-IX treatment. Representative pictures are shown at 40x magnification.

The increased expression of p21 taken together with increased phosphorylation of p53 was indicative for DNA. We therefore analyzed phosphorylation of the histone variant H2A.X as a marker for DNA double stand breaks. In all cell lines, especially in BxPC-3 and MIA PaCa-2, increased phosphorylation was detected in immunocytochemistry (**Figure 8**).

Taken together, these results indicate that JNK-in-IX is effective in PC through reduced cell survival and restrained cell migration. These effects are mediated through DNA damage resulting in a G2 arrest and incapability to enter mitosis through activation of p53 and p21 independent of JNK2.

## Discussion

PC is continuously proving itself as one of the most challenging malignancies to detect and treat. Recent efforts in systemic treatment options had two main goals: large, multicenter studies aimed at determining the optimal chemotherapies for whole patient cohorts (24, 25). On the other hand, efforts were made in personalizing treatment options using novel modeling systems like organoids (26). One of the major obstacles in drugging PC is the fact that its major driver, mutant KRAS, has presented itself undruggable (4). Furthermore, it has been shown that mutant KRAS causes alterations in associated pathways, thereby potentiating its deadly potential (27). However, we believe that targeting these associated pathways may be an attractive treatment option.

The c-Jun N-terminal kinases (JNK)1, 2, and 3, together with p38 kinases, are also called stress activated kinases as they are involved in the cellular response towards exogenous and endogenous stressors like metabolic stress or cytokine stimulation, as well as UV radiation or cytotoxic drugs (6). Due to their central role in the cellular signaling cascade they merge signals from multiple membrane receptors and intracellular kinases including RAS. Multiple substrates of JNK have been identified and include transcription factors like c-Jun and p53, apoptosis regulating proteins but also cytoskeleton elements (6).Previous studies have implicated a potential role of JNKs in PC (11, 13).

In determining the roles of JNK in PC, several factors need to be considered: isoform specific roles of JNK1 and 2 need to be taken into account, while JNK3 is only expressed in brain, heart and testis. Additionally, as demonstrated by Sato and colleagues (11), the overall role of JNK in PC also includes the kinase action in cells of the tumor microenvironment. By silencing JNK1 and 2 separately, we recently demonstrated a tumor restraining function of JNK2 and a tumor promoting role of JNK1 (18). These results prompted us to investigate the value of isoform specific JNK inhibitors for the treatment of PC.

We used four pancreatic cancer cell lines with different genetic backgrounds, including KRAS-wildtype BxPC-3 (28). All cell lines were treated with JNK inhibitors addressing different backgrounds: SP600125 is a commonly used reversible ATP-competitive pan-JNK inhibitor (29) which effectiveness in PC was shown by us and others (18, 30). AS602801 (Bentamapimod) also acts as an ATP competitive inhibitor with similar IC50 values for JNK1 and 2. Interestingly it was shown effective against cancer stem cells including cell survival, as well as self-renewal and tumor-initiating capacity in PC (31). Contrary to those, Licochalcone A is not ATP-competitive but competes with the scaffolding protein JIP1 in its binding with JNK and thereby inhibits specifically JNK1 activity (32). Lastly, we used the JNK2 specific, ATP competitive JNK inhibitor IX (JNK-in-IX) (33). We can now demonstrate that both pan-JNK inhibitors as well as the JNK1 specific inhibitor exert similar growth restraining effects across most cell lines. However, JNK-in-IX has proven itself as the most effective by far of all inhibitors and was even effective in PANC-1 which consistently shows resistance against JNK inhibition (18, 34). Although less pronounced, JNK-in-IX was also effective in primary PC organoids, underlining the potential inhibitor use. These interesting findings were opposing to our previous demonstration of a growth suppressive function of JNK2 and prompted further studies.

Being stress-activated kinases, JNKs are involved in the cellular response towards endogenous and exogenous stressors. Therapy-induced cell stress through radiation or cytotoxic stress is a fundamental part of successful cancer treatment. By inhibiting the cellular coping mechanisms to therapy induced stress, therapy resistance might be overcome. We therefore decided to evaluate the combination treatment of the most effective JNK-in-IX and the standard cytotoxic treatments for PC, Gemcitabine-Paclitaxel (Gem-Pac) and FOLFIRINOX. Contrary to Lipner and colleagues who showed sensitization of PC cells towards 5-FU/FOLFOX treatment after JNK1 inhibition (19), JNK-in-IX did not show treatment synergy with FOLFIRINOX or Gemcitabine-Paclitaxel.

As the invasion ability is a hallmark of cancer cells, we next examined the effectiveness of JNK-in-IX on reducing cell migration *in vitro*. Again, wound closure of all cell lines was significantly inhibited. More importantly, the seen effect was even more pronounced in cell migration. Recently, Jemaà and colleagues used JNK inhibition in order to reduce colon cancer cell migration. Interestingly, they were able to reduce migration of human RKO cancer cell line *in vitro* by pan-JNK inhibition and specific JNK1 inhibition. However, JNK-in-IX was ineffective (35). On the other hand, the group of van Berg demonstrated the value of JNK2 for breast cancer cell migration (36) and pharmacological JNK2 inhibition reduced cell migration (37).

In our study, the reduced cell proliferation and ultimately cell death was apoptosis independent. Furthermore, we were able to show that the seen effects are caused by a G2/M arrest. G2/M progression is dependent on the active complex of CDK1 and Cyclin B1. CDK1/Cyclin B1 phosphorylation inhibits the complex pro-mitotic activity and is mediated through Wee1. In contrast, the phosphatase CDC25C activates CDK1/Cyclin B1 through dephosphorylation. Other regulators of cell cycle progression act through activation (PLK1) or deactivation (CHK1) of CDC25C (38). Finally, p53 can repress CDK1 and CCNB1 expression and additionally inhibit CDK1 directly through activation of p21 which binds and inhibits CDK1 directly (39).

In the present study, we demonstrate that JNK-in-IX leads to a G2 arrest in pancreatic cancer cells *via* increased expression of p21 (CDKN1A). Together with the increased phosphorylation of p53, as demonstrated in the proteome profiling, this suggested that the inhibitor treatment may result in DNA damage. We were able to confirm increased DNA double stand breaks through ICC labeling of Histone H2A.X phosphorylation.

Similar findings have been demonstrated for JNK-in-IX in Jurkat T-cells (40). Although in this lymphoid cell line, cell death was due to apoptosis, cells also underwent a G2 arrest. Furthermore, JNK-in-IX treatment resulted in a defective mitotic spindle defect (40). This mechanism could serve as an explanation for the morphological differences observed in our treatment group.

The findings of our present study indicate JNK inhibitor IX as a promising therapeutic in PC. However, our present findings were in contrast to previous studies from our group: Stable knockout of JNK2 increased proliferation and migration of PC cells (18). Thus, we examined the effectiveness of JNK-in-IX in inhibiting the JNK2 activity. Interestingly, c-Jun phosphorylation as major downstream target of Jun kinases was unaltered. Furthermore, siRNA-based knockdown of JNK2 did not impair the effectiveness of JNK-in-IX. These findings indicate that JNK-in-IX may act independently of JNK2, at least in the studied cell lines.

Overall, our study underlines the current trend in personalizing therapies. JNK2 as the primary target of the compound used in our study did not seem to be promising based on genetic findings but the way of action of JNK-in-IX was proven effective. Novel model systems like tumor organoids (41) can test these compounds and thus be a powerful tool in prioritizing treatment options and verify or disprove treatment options based on genetic testing.

## Data availability statement

The original contributions presented in the study are included in the article/**Supplementary Material**. Further inquiries can be directed to the corresponding author.

## Ethics statement

The studies involving human participants were reviewed and approved by Independent Ethics Committee of the University of Ulm. The patients/participants provided their written informed consent to participate in this study.

## Author contributions

Conceptualization, MK and BT; Literature Search, XY, QK, MD, MK, and BT; Experiment and Data Analysis, JS, XY, QK, JL, and MD; Writing – JS, MK, and BT; Supervision, BT. All authors read and approved the final version of the manuscript.
