# Supplementary figures and images for: JNK inhibitor IX restrains pancreatic cancer through p53 and p21
Source: Front Oncol. 2022 Dec 7;12:1006131. doi: 10.3389/fonc.2022.1006131 (PMC9768178; doi:10.3389/fonc.2022.1006131)

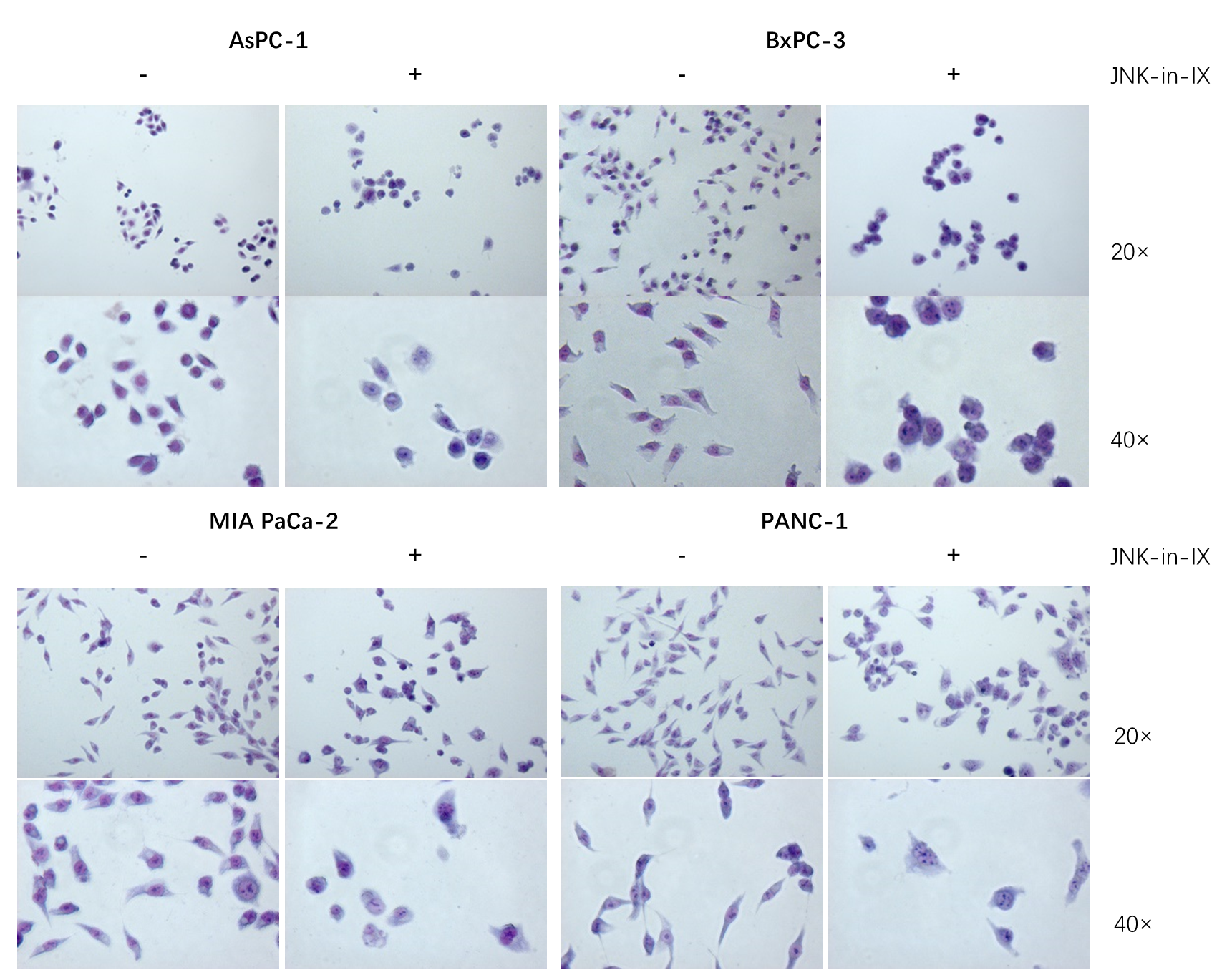

Supplement: Supplementary file 1 [file Image_1.tif]

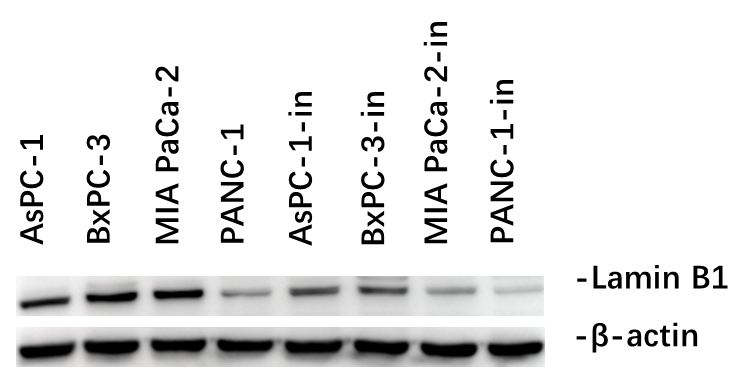

Supplement: Supplementary file 2 [file Image_2.tif]
