# Supplementary material for: JNK inhibitor IX restrains pancreatic cancer through p53 and p21
Source: Front Oncol. 2022 Dec 7;12:1006131. doi: 10.3389/fonc.2022.1006131 (PMC9768178; doi:10.3389/fonc.2022.1006131)
Supplement: Supplementary file 3 [file Table_1.docx]

| **Gene name** | **primer** | **Catalog** | |
| --- | --- | --- | --- |
| **GAPDH** | Hs_GAPDH_1_SG | | Cat.NO.QT00079427 |
| **CDK1** | Hs­­­_CDK1_1_SG | | Cat.NO.QT00042672 |
| **CCNB1** | Hs_CCNB1_1_SG | | Cat.NO.QT00006615 |
| **CDC25C1** | Hs­­­_CDC_25C_1_SG | | Cat.NO.QT00000350 |
| **PLK1** | Hs_PLK1_1_SG | | Cat.NO.QT00049749 |
| **CHEK1** | Hs_CHEK1_1_SG | | Cat.NO.QT00006734 |
| **CDKN1A,** | Hs_CDKN1A_1_SG | | Cat.NO.QT00062090 |
| **p53** | Hs_TP53_1_SG | | Cat.NO.QT00060235 |
| **Wee1**  **MAPK8**  **MAPK9** | Hs_WEE1_1_SG  Hs_MAPK8_1_SG  Hs_MAPK9_1_SG | | Cat.NO.QT00038199  Cat.NO.QT00091056  Cat.NO.QT00069909 |

**Supplementary Table S1.** Primers used in qPCR.
