# Supplementary material for: JNK inhibitor IX restrains pancreatic cancer through p53 and p21
Source: Front Oncol. 2022 Dec 7;12:1006131. doi: 10.3389/fonc.2022.1006131 (PMC9768178; doi:10.3389/fonc.2022.1006131)
Supplement: Supplementary file 4 [file Table_2.docx]

**Supplementary Table 2.** IC50 values (μM) for JNK inhibition of human pancreatic cell lines AsPC-1, BxPC-3, MIA PaCa-2 and PANC-1 respectively for SP600125, AS602801, JNK-in-IX and Licochalcone A.

|  | **SP600125** | **AS602801** | **JNK-in-IX** | **Licochalcone A** |
| --- | --- | --- | --- | --- |
| **AsPC-1** | 38.10 | 17.21 | 0.41 | 7.40 |
| **BxPC-3** | 7.64 | 9.31 | ~ 0,22 | ~ 13,85 |
| **MIA PaCa-2** | 11.87 | 6.45 | 0.07 | 13.48 |
| **PANC-1** | Nd ^1^ | ~ 16,66 | 0.07 | ~ 16,92 |

^1^ Not determined: Due to intrinsic resistance of PANC-1 towards SP600125 treatment, IC50 values couldn’t be determined.
