# Supplementary material for: JNK inhibitor IX restrains pancreatic cancer through p53 and p21
Source: Front Oncol. 2022 Dec 7;12:1006131. doi: 10.3389/fonc.2022.1006131 (PMC9768178; doi:10.3389/fonc.2022.1006131)
Supplement: Supplementary file 5 [file Table_3.docx]

**Supplementary Table S3.** Zero Interaction Potency (ZIP) Score of AsPC-1, BxPC-3, MIA PaCa-2, PANC-1 treated by chemotherapeutic drugs and JNK-in-IX.

|  | Gem-Pac/JNK-in-IX | | | FOLFIRINOX/JNK-in-IX |
| --- | --- | --- | --- | --- |
| AsPC-1 | | -6.55 | -13.81 | |
| BxPC-3 | | -2.32 | -3.27 | |
| MIA PaCa-2 | | -0.61 | -2.38 | |
| PANC-1 | | -6.08 | -1.95 | |
